# Wake-up call for recovery: a paradigm shift to address the deep crisis in Israel’s public mental health services in the shadow of October 7, 2023

**DOI:** 10.1186/s13584-025-00670-y

**Published:** 2025-01-29

**Authors:** Amir Krivoy, Gadi Rosenthal

**Affiliations:** 1 Geha Mental Health Center, Helsinki 1st, Petach-Tikva, +9729258220 Israel; 2 Mental Health Research Center, Clalit, Petach-Tikva, Israel; 3 School of Medicine, Faculty of Medical and Health Sciences, Tel-Aviv University, Ramat-Aviv, Israel

## Abstract

**Background:**

The events of October 7, 2023, and the subsequent war have starkly exposed the shortcoming of Israel’s public mental health system. This system, already strained by years of underfunding and the COVID-19 pandemic, was unprepared for the surge in mental health needs resulting from these traumatic events. This paper outlines the systemic failures and proposes a comprehensive overhaul reform towards an integrative community-based, recovery-oriented mental health service.

**Main body:**

Israel’s mental health crisis is exacerbated by four converging vectors: a global diagnostic crisis in psychiatry, insufficient biological treatments, chronic underfunding, and a fragmented service model. Diagnostic practices, centered on outdated classifications, fail to address the complexity of severe mental illnesses, resulting in imprecise diagnoses and insufficient treatments. Despite the advent of psychopharmacology, significant advancements in drug efficacy are lacking, with recovery rates stagnating or declining. Financially, mental health in Israel receives only 5.2% of the health budget, far below the 10–16% seen in high-GDP Western countries. The community mental health services reform in 2015 lack effective oversight and incentives, leading to long wait times and inadequate care. Additionally, the fragmentation among funding entities—HMOs, Ministry of Health, and Ministry of Welfare—hampers coordinated care and comprehensive service delivery.

**Conclusion:**

The proposed solution involves shifting from a hospital-biomedical -based to an integrated community-based model, emphasizing recovery over symptom management, based on regional mental health centres as hubs of services. This requires significant investment in community mental health teams, crisis intervention, home treatment, and integrated services. Early intervention, technology utilization, economic incentives for community-based care, and patient and family involvement are crucial components. This transformation aims to create a holistic, efficient, and patient-centered mental health system, better equipped to handle future challenges and reduce the societal and economic burdens of mental illness in Israel.

## Background

The events of October 7, 2023, and the ensuing war confronted a fragmented, depleted, and collapsing Israeli public mental health system [1]. The availability of services and the ability to provide a reasonable response do not meet the needs in all dimensions. The grim state of the public mental health system even before recent events results from a combination of negative consequences of several global and local processes, leading to chronic underperformance and inability to provide quality responses in a reasonable timeframe, as expected from a Western health system and according to the Israeli National Health Insurance Law [2]. The system has been in chronic insufficiency for years, and the past four years added the impact of the pandemic and its challenges (lockdowns, isolations, tests, unemployment, etc.), leading to a steep increase in mental illness rates and demand for mental health services, while the response remained inadequate. The traumatic events following the October 7th war and their impact on various publics’ mental health are still being studied, and it is likely we will see their long-term effects in the coming years [3].

As a result, the growing consumer public remains without adequate community responses, and thus, the primary solution available is the use of the hospitalization system. Psychiatric emergency room visits are free of charge for the patient in Israel [4], often leading to unnecessary hospitalizations simply because it is the most readily available solution for diagnosis and mental treatment in the absence of appropriate alternatives for intensive care and crisis solutions.

Families of the mentally ill also pay a heavy price. This manifests in an endless investment of mental resources, time, and money, often leading to significant suffering. Beyond their own post-traumatic crises linked to the crises of their mentally ill kins, these families also face a lack of systemic support. Approximately 300,000 people in Israel [5] suffer from severe mental illness (Severe Mental Illness - SMI), with another 700,000 family members needing support and assistance. In total, this amounts to one million people, or 10% of the population. Additionally, there are an estimated 1.25 million people requiring outpatient treatment (milder mental illness). Altogether, about 2.25 million people, close to a quarter of the population, are estimated to be affected. In the near future, we will need to add thousands more people who will suffer from long-term post-traumatic syndromes and/or worsening mental illnesses due to the war events. These new patients and their families will have different demographic characteristics, necessitating tailored treatments.

The mental health landscape encompasses a wide spectrum of conditions, ranging from severe mental illnesses (SMI) to milder, non-SMI conditions. It is crucial to recognize that these different categories require distinct treatment approaches, resource allocation, and support systems.

SMI typically includes conditions such as schizophrenia, bipolar disorder, and severe major depression. These conditions often require intensive, long-term care, ongoing, comprehensive care that may include medication management, psychotherapy, and social support. A multidisciplinary team approach that often involves psychiatrists, psychologists, social workers, and occupational therapists working in concert. Focus on functional recovery: While symptom management is important, there’s a strong emphasis on improving overall functioning and quality of life. Family involvement: Families often play a crucial role in the care of SMI patients and may require support and education themselves. Community-based services: Assertive Community Treatment (ACT) teams and other intensive community support programs are often beneficial and crisis intervention services are crucial to manage acute episodes and prevent hospitalizations.

Non-SMI Conditions include milder forms of depression and anxiety disorders, adjustment disorders, and other less severe mental health issues. Many non-SMI conditions respond well to time-limited treatments, such as brief psychotherapy or short-term medication courses. Many non-SMI conditions can be effectively managed within primary care settings, with support from mental health specialists as needed. Evidence-based psychotherapies such as Cognitive Behavioral Therapy (CBT), Interpersonal Therapy (IPT), and other evidence-based approaches are often effective for non-SMI conditions. Many individuals with non-SMI conditions benefit from self-help resources, online therapy platforms, and mental health apps.

Post-Traumatic Stress Disorder (PTSD) occupies a unique position in the mental health spectrum. While it can be severe and chronic, it also responds well to specific, time-limited interventions. Evidence suggests that early, targeted interventions can prevent the development of chronic PTSD. Specialized treatments such as Trauma-focused CBT and Eye Movement Desensitization and Reprocessing (EMDR) have shown high efficacy for PTSD. Unlike many SMIs, PTSD can often be effectively treated with relatively short-term interventions, potentially avoiding progression to chronic states. PTSD often co-occurs with other conditions (both SMI and non-SMI), requiring careful assessment and tailored treatment plans.

Recognizing these differences has important implications for mental health system design:

Resource allocation: While SMI requires significant long-term resources, investing in accessible, short-term interventions for non-SMI conditions can prevent escalation and reduce overall system burden. Workforce development: The system needs a diverse workforce, from highly specialized SMI treatment teams to primary care providers trained in managing common mental health conditions. Service continuum: A well-designed system should offer a continuum of services, from brief interventions in primary care to intensive community-based programs for SMI. Family support: While crucial for SMI, family support and education can also be beneficial for non-SMI conditions, albeit often in different forms. Measurement and outcomes: Different outcome measures and treatment durations are appropriate for SMI versus non-SMI conditions, necessitating flexible approaches to quality assessment and value-based care.

The Israeli society pays a very high price for neglecting mental health. The total value of mental health problems in OECD countries is estimated at 4–6% of GDP [6]. The impact of COVID-19 further increased this percentage. Reducing this impact by 1% could save Israeli society about $22 billion, approximately five times the total public expenditure on mental health.

Israel’s mental health system is deeply rooted in the bio-medical-hospital model, with significant gaps in community-based services. These gaps include insufficient assertive community treatment teams, limited crisis intervention services, inadequate access to psychotherapy, scarce step-down services, and underdeveloped triage systems. This comprehensive deficit in community-based care has resulted in over-reliance on hospital-based services as the default solution for mental health crises. Hospitals, inpatient units and mental health outpatient clinics are managed by physicians, and treatment success is measured by the ability to eliminate symptoms, cure the illness, and return to full functionality. In such metrics, considering the significant difficulties in treating severe mental disorders, successes are infrequent, the challenge is immense, and it is easy to become frustrated and despairing for both patients and their families and the medical staff.

## Main text

The current deep crisis in the health system and the enormous and growing need also present an opportunity for a comprehensive revolution and overhaul in building Israel’s mental health system, beyond one-time financial investments that serve as an inefficient “band-aid” solution. Israel’s mental health service must transition to a community-based, recovery-oriented model, extending from the biomedical model to a regional based whole-service approach. For this, a comprehensive plan with a realistic investment amount is required to build and maintain a service available to the growing demand. Below, we will briefly describe the components of the crisis and the program for the required solution:

### Four vectors converging to impact the disastrous outcome of Israel’s mental health system: two global and two local

#### 1. Global diagnostic crisis in psychiatry

The existence of chronic brain diseases manifesting in significant mental symptoms is unquestioned. However, the attempt to group symptoms into disorders in the hope of finding a biological origin and specific treatments, as done by the DSM and ICD over the past decades, has failed [7]. This is particularly evident in more severe mental disorders, where diagnoses like schizophrenia, bipolar disorder, or depression are not precise enough [8]. Despite decades and billions of dollars invested in research, diagnosis and treatment of people with severe mental illness have not improved. Many symptoms can belong to multiple diagnoses, leading to situations where one person might have four or five different diagnoses. Conversely, there are symptom clusters that do not match any specific diagnosis according to the DSM or ICD. In Israel, eligibility for mental health services and other derived benefits depend on category-based diagnoses. Moreover, to diagnose, a doctor is required, hence the system relies on medical personnel.

#### 2. Crisis of biological treatments in psychiatry

The onset of the psychopharmacological era in the 1950s heralded great promise for treating mental illnesses and aligned with the biomedical model that mental health has leaned towards ever since. In the years since, and especially in the last 30 years, no breakthrough has been recorded in drug treatment for brain diseases manifesting in mental symptoms. Moreover, as high-quality, unbiased clinical research data accumulates, the very limited impact of drug treatment on the recovery ability of people with severe mental illness becomes clearer [9]. Studies examining recovery rates over different decades have shown that not only do recovery rates not change, they even decrease over time. Psychiatric drugs are non-specific; for example, antidepressants are used to treat depression, anxiety, obsessive-compulsive disorders, and more. Additionally, drugs do not cure diseases but are symptom-oriented only, and even that, in a limited manner. Israel’s mental health system is based on drug treatment and prioritizes biological medical treatment. As a result, the availability of non-drug treatments, such as psychotherapies and social treatments, is relatively low and does not form the core of the system.

#### 3. Crisis of resources in Israel’s mental health

There is a chronic lack of budget since mental health is traditionally considered the “backyard” of health in Israel. Our recent calculation showed [10] that the share of public expenditure on mental health (i.e. all treatment modalities in mental health facilities in the community or hospitals and at rehabilitation) out of total health expenditure in Israel was 5.2% in 2021 data. While in high-GDP countries, of which Israel is in the upper half, the rate is around 10%, and in Western European countries, even 12-16%. Despite transferring responsibility for community mental health care to the HMOs in July 2015, no appropriate supervision and incentive mechanisms were built to create an efficient and available community service. Waiting times for psychological and psychiatric diagnostic appointments are unreasonable. According to a 2021 report by the Myers-JDC-Brookdale Institute in Israel, the average waiting time for a first appointment with a child psychologist in Israel’s public health system is 4.5 months. The median waiting time is even longer − 6 months. This is an unreasonable waiting time, especially for children and youth who require timely intervention [11].

Another example is the budget al.located to mental health from the drug and technology basket, less than 1% over the years (partly due to the lack of new technologies in mental health). This is minimal and does not represent the proportion of mental illness in the overall burden of disease in the public (about 20%). Furthermore, hospital funding is based on “dumb” money, blind payment per hospitalization day without considering the quality of treatment in hospitals and the resources invested in patients there. While value-based treatment incentives are forming worldwide [12], Israel’s regulator fails to persuade HMOs to invest in mental health patients through financial support incentives. Since HMOs are not obligated, they are usually uninterested (or unable) to develop such a service. Moreover, there is a significant shortage of professional staff, particularly a shortage of psychiatrists (especially child and adolescent psychiatrists), with an expected deficit of hundreds in the coming years. Unfortunately, the system in Israel is currently built so that without psychiatrists, it is impossible to build psychiatric wards or clinics. Additionally, in the last two years, there has been an increasing transition from the public system of mental health professionals such as social work and psychology due to low compensation and hard, wearing work in the public sector compared to conditions in the private sector.

The result is a significant expansion of the private service with significantly higher tariff levels than other medical branches. Families’ direct annual expenditure on their loved ones dealing with mental illness is 50% higher than the total public expenditure on mental health. The economic outcome is harsh: while in general medicine in Israel, 63% is public expenditure, and 37% is private expenditure (a result not excellent in itself [10], in mental health, the proportions are more or less reversed, meaning families spend most of their mental health expenditure out of their pockets. Needless to say, these parameters’ implications for inequality and social welfare are significant. This is in direct opposition to the spirit and purpose of the National Health Insurance Law.

### Note on the impact of the war on resource and professional workforce issues

The large population group that will suffer from trauma (according to existing knowledge from mass traumas, only a part, not the majority, will become chronically post-traumatic) will create an urgent need for training trauma therapists and allocating resources to the issue. It is important to ensure that this important resource allocation does not come at the expense of or delay treatment for existing system problems. The health, social, and economic costs of such a priority order will be very high and extreme. Both important needs must be addressed simultaneously.

#### 4. Service crisis in Israel’s mental health

The primary response in Israel’s mental health is heavily based on the hospitalization system, while the community response is ambulatory, meaning passive and reactive, waiting for the patient to schedule an appointment and meet with the doctor or therapist. The service in the community is often disconnected from the hospitalization and does not form an essential link in the treatment continuum. There is built-in fragmentation in the system between hospitals and the community, between psychiatric hospitals and the general medical system, and between rehabilitation services and mental health treatment services [13]. The current treatment approach gives excessive emphasis (also budgetary) to hospitals and hospitalization beds. This, while the structural reform of 1995 led to a decrease in the hospitalization bed rate, dropping by more than 50% in the last two decades [14]. Due to the lack of a true treatment continuum in the community and the paucity of a community-based service, there is a lack of focus and clear, sustainable, and achievable treatment goals, resulting in unreasonable loads on a depleted and neglected hospitalization system and unreasonable hospitalization conditions in psychiatric hospitals.

### Additional prominent fragmentation in the system’s economic-financial structure

The mental health system in Israel is financially fragmented among three entities:

The HMOs, which have been funding intensive treatment systems (mainly hospitals) and outpatient systems since the 2015 insurance reform.

The Ministry of Health, which directly funds the community rehabilitation system through tenders to service providers.

The Ministry of Welfare, which owns and funds part of the rehabilitation units (particularly relevant to large groups of dual-diagnosis, multiple disabilities, children, and adolescents).

This fragmentation and discontinuity lead to a lack of overall vision, particularly to autonomous incentive systems that do not see the overall well-being of mental health patients and their families. The Community Mental Health Rehabilitation Law (2000) is progressive, promising a rehabilitation basket to all mental health patients [15, 16]. In practice, 24 years after its enactment, it serves only 37% of those recognized as disabled based on mental health and about 12% of all patients [14, 16]. Additionally, there has been a delay of about two decades in issuing housing solutions, which constitute about 60% of the total expenditure within the law.

### Noteworthy progress components

The system has components of progress: the beginning of establishing “stabilizing houses” or “balancing homes” (a community-based mental health facility that provides an alternative to traditional psychiatric hospitalization, based on the Soteria model), the start of removing chronic patients from hospitals to care homes in the community, family centers, etc., and a certain degree of problem recognition. However, these are insufficient to advance Israel’s mental health system to a quality and beneficial system.

The solution to all these crises and the only way to change the negative direction in which the system is heading is a strategic plan whose compass and leading vision is the rebuilding of the system so that each of its components and its total parts are directed to community-based recovery treatment. These simple words have deep and practical meaning. Changing the treatment focus, the purpose of the system’s therapeutic action, from cure (medical model) or symptom improvement to the recovery model, has significant power and implications at the individual, family, and overall health system levels.

### Transition from medical model to recovery model and building a recovery-based service

Transitioning from a medical model to a recovery model and building a recovery-based service is the appropriate solution for Israel’s mental health system, which needs a significant shake-up. The recovery movement [17]began with the initiative of consumers, patients, and their families and has gained widespread acceptance in many health systems, including the UK, Australia, the Netherlands, the United States, and more [18].

In the recovery approach, great emphasis is placed on the personal, individual journey of patients to find meaning and value in life while dealing with mental challenges, with the family being part of the journey, empowering and empowered. This perspective aims for success and functioning in the community alongside the symptoms, rather than symptom elimination and recovery from illness as a goal. Key principles include: Personhood: Viewing the individual as a person, not just a collection of symptoms or a diagnosis. Self-direction: Empowering individuals to lead their own recovery journey. Holistic approach: Addressing all aspects of a person’s life, not just their mental health symptoms. Non-linear process: Recognizing that recovery is not a straightforward path but may involve setbacks and progress. Peer support: Valuing the role of lived experience and mutual support in the recovery process. Respect: Accepting and appreciating individuals as they are, including their experiences and autonomy. Responsibility: Encouraging individuals to take responsibility for their own recovery. Hope: Maintaining a belief in the possibility of a fulfilling life, despite mental health challenges.

This perspective aims for success and functioning in the community alongside the symptoms, rather than symptom reduction and recovery from illness as a goal. In the recovery approach, diagnoses are not central; rather, the severity of symptoms and the functional difficulty arising from them are. Additional principles in a recovery-based service include connecting to the healthy aspects, viewing the person as a whole rather than a collection of symptoms, empowering and strengthening personal skills, and social connection to the community. Implementing a recovery-based model is expected to lead to several positive outcomes: Improved quality of life, increased community integration, reduced hospitalizations, enhanced self-management, improved relationships with family involvement and a focus on social connections, reduced stigma, and cost-effectiveness on the long-term.

While expanding beyond the traditional medical model, the proposed system maintains and integrates essential medical expertise within a broader recovery-oriented framework. This integration acknowledges the vital role of medical knowledge and interventions while addressing the limitations of a narrowly focused medical approach. The expanded model combines clinical expertise with psychosocial interventions, peer support, and community integration, creating a more comprehensive approach to mental health care that better serves the full spectrum of patient needs.

It is pivotal to measure recovery-oriented outcome using established tools like the Recovery Assessment Scale (RAS) or the Mental Health Recovery Measure (MHRM) which can assess individual progress in recovery. Also, functional outcomes such as measures of employment, education, housing stability, and social engagement can indicate community integration.

While the dominance of hospital-based care and the limitations of the narrow medical model are interrelated, they represent distinct challenges. The hospital-centric nature of the system reflects structural issues in service delivery and resource allocation, while the limitations of the medical model relate to conceptual approaches to mental health care. Despite significant reductions in psychiatric bed numbers over the past decades, hospitalization remains the dominant solution due to insufficient community alternatives rather than therapeutic necessity. The solution involves both developing robust community-based services and expanding the medical model to incorporate recovery-oriented principles.

### Recovery-based mental health system

A recovery-based mental health system is built to provide a personalized, patient-based response with varying intensity according to the patient’s and their family’s needs and challenges during the journey. Such a service must include components of continuous response, aiming to keep treatment in the community as much as possible and avoid hospitalization. If hospitalization is necessary, it should be as short as possible and support the best return to the community. Therefore, significant resource investment in multi-professional community teams is crucial, as is establishing an intensive service model for assertive community treatment and significantly strengthening complementary services such as home treatment, community crisis intervention teams, high availability of individual and group psychotherapies, peer support groups, family treatments, and more. The main idea is to surround patients with a coordinated personal framework until entering recovery - meaning returning to the community and living with meaning - understanding that this is a journey that can take time and is usually not linear, with setbacks. The model’s focus on functional outcomes and subjective well-being is equally relevant for both SMI and non-SMI populations, though the specific interventions and intensity of services will differ based on individual needs. People with milder mental illness may benefit from multiple entry points with lower barriers to access, early intervention services to prevent progression to more severe conditions, integration with primary care for milder conditions and flexible service intensity that can be adjusted based on individual needs.

There are alternatives redesign approaches, such as strengthening the existing biomedical model by improving and expanding the current hospital-centric, biomedical model of mental healthcare. This could involve increasing funding for inpatient facilities, enhancing psychiatric medication research and development, and optimizing the use of psychopharmacology. Other option may be integrating mental health into primary care by placing a stronger emphasis on integrating mental healthcare into primary care settings. This would involve upskilling general practitioners and equipping them with the resources to manage a broader range of mental health conditions.

While each of these alternative options has its merits, we believe the community-based, recovery-oriented model is the most comprehensive and appropriate solution for Israel’s mental health system. Our rationale is as follows: a holistic, person-centered focus, addresses the full spectrum of the patients’ needs. This is a more effective approach than the narrower, symptom-focused biomedical model. Moreover the recovery model has been shown to lead to better outcomes in terms of community integration, employment, and overall quality of life for individuals with mental health challenges. And lastly the recovery model can be effectively applied to a wide range of mental health conditions, from severe mental illnesses to milder disorders, as well as specific conditions like PTSD.

Addressing System-Level Challenges: By focusing on regional integration, continuity of care, and innovative workforce and financing models, the proposed reforms address the systemic issues that have plagued Israel’s mental health system, going beyond simply enhancing specific treatment modalities (see Table 1 for a summary of problems in the Israeli mental health system and suggested solutions).

While the alternative options have their merits and may play a complementary role, we believe the comprehensive, recovery-oriented model offers the best path forward for transforming Israel’s mental health system and improving outcomes for individuals, families, and the broader society.

Psychiatric rehabilitation is essentially a comprehensive system approach to promoting recovery-based service. The construction of the treatment and support plan in a rehabilitative framework is the initial therapeutic approach in the assessment meeting. Thanks to the miracle of the Mental Health Rehabilitation Law, the rehabilitation system in Israel is extensive and enjoys stable annual funding despite the mentioned difficulties. However, building a plan for rehabilitating the entire mental health response is necessary, with the rehabilitative response forming the main path to which the recovery journey will lead, combined with the therapeutic arm. Such a change in the system will entail additional changes such as strengthening patient autonomy, reducing the covert paternalism in the system, using shared decision-making mechanisms, and giving patients a voice as part of the therapeutic team. More than anything, such a transition will allow everyone involved in mental health, both patients and therapists, much more optimism and hope, so necessary in the face of complex challenges.

### Proposed principles for a program to rebuild a recovery-based mental health system

#### The regional-integrational reform

Transition from a hospital-based structure to comprehensive treatment zones (regional mental health centers) that will provide a unified and continuous service to patients in their geographical area. The regional service will include a hospitalization component (based on existing hospitals but smaller) and an extensive community component that includes the full treatment intensity continuum. The regional service director will be chosen based on managerial-professional skills. The same manager may be the regional hospital director. Their role will be to build the integrational service array in the area according to the relevant needs, seeing an incentive system that benefits all patients and families. The fact that both the psychiatric hospital system and the community system of the HMOs are already based on regional anchors should facilitate the transition to a regional structure. It should be noted that a pioneering attempt of this kind was made to some extent with the outbreak of the war events, initiated by the Ministry of Health when the mental health response in the various war evacuees’ hotels across the country was divided on a regional basis to the responsibility of hospitals for building teams and operating them according to the relevant challenges of each area.

1. In such a structure, even during psychiatric hospitalization (most of which should be reserved for acute exacerbations or involuntary admissions ), the responsibility for the patient’s treatment continuum will remain with the community teams, coordinating with the intensive treatment team, to shorten the hospitalization period as much as possible and enable the construction of appropriate support systems (therapeutic and rehabilitative) as quickly as possible in the community, combined with local support factors (local councils, NGOs, etc.).
2. The community service should be built as a continuum according to the intensity of resources invested in treatment: full hospitalization -> day treatment -> balancing home -> home treatment -> assertive community treatment -> mental health clinic treatment -> primary community clinic treatment. The transition of patients on the continuum will be carried out by the function of treatment coordinators (not currently existing), who can come from all relevant health professions. A continuous envelope with varying intensity will be based on diverse mental health responses and tailored thoughtfully to each patient according to their condition at any time. The HMOs, currently responsible for the community mental health provision, will be able to purchase services from the region for their members or from other providers in the area, thus maintaining a market with competition for the benefit of the insured.
3. A recovery-based system will lead to psychiatric diagnoses not being a barrier to entering the treatment system, given the low validity of psychiatric diagnosis (as explained above). Accordingly, the system structure will allow for a variety of entry points (based on symptoms and function rather than formal diagnoses), available, accessible, and efficient, enabling early detection and entry into a focused recovery process. Entry points can be through local community services (welfare, health, and education), through primary care in the community (family doctor), or through direct contact (hotlines, virtual response, or face-to-face) to the mental health system.
4. There is a need to reverse the workforce pyramid – primarily relying on therapists in disciplines that require less training time to provide initial response and triage (screening) to transition to an expert level (psychiatry, psychology). Since the initial response is based on psychosocial interventions, increasing the immediate workforce will allow for quicker therapeutic contacts and the ability to screen appropriate cases only for the expert level (which has been decreasing in quantity over the years). Using similar treatment models worldwide, such as IAPT, to increase the workforce for initial contacts in the community. In light of what has been described above, returning private system therapists to the public system will involve significantly increasing financial incentives and creating meaningful roles with promotion prospects.
5. Building a specialized services for early diagnosis and early intervention in young ages to start early treatment (multi-disciplinary) and entry into the recovery process (personal and family) at early stages (reducing the transition to chronicity later -> reducing cumulative cost).
6. Using technology as a tool to fill workforce gaps (which will only worsen over time), the geographical distribution issue, and accurately measuring the therapeutic value of interventions, data-based and personalized.
7. Using relevant and appropriate economic levers to encourage patients’ transition to community treatment and quick discharge from hospitalization and using the regulator’s ability to push HMOs to build the extensive community services as detailed above. Transitioning to value-based therapeutic compensation according to clear metrics that will be set based on the resources invested by the HMOs in providing the service. Although it is a challenge many health systems face as there is very little value-based metrics for measuring outcomes in mental health [19, 20].
  Implementation of value-based payment mechanisms is crucial for incentivizing appropriate care delivery. This includes:
  –Developing specific quality metrics for community-based care that reflect recovery-oriented outcomes.
  –Creating financial incentives for HMOs based on: successful implementation of comprehensive community services, reduction in avoidable hospitalizations, achievement of specific recovery-oriented outcomes, integration of services across the care continuum, implementing a balanced incentive structure that rewards both quality of care and efficient resource utilization and establishing clear accountability measures for service delivery and outcomes.
  The payment structure should explicitly reward HMOs, for investing in prevention, early intervention, and community-based services, while maintaining appropriate access to intensive services when needed. This represents a shift from the current volume-based payment system to one that emphasizes value and outcomes in line with the recovery model targets.
  Any incentives related to hospitalization length must be carefully designed to promote appropriate rather than simply shorter stays. These incentives should be implemented only after establishing comprehensive community support services, include quality metrics that monitor readmission rates and patient outcomes, recognize that some patients, particularly those with severe mental illness, may require longer periods of high-quality inpatient care, balance the goals of efficient resource utilization with optimal clinical outcomes and include mechanisms to prevent premature discharge and the ‘revolving door’ phenomenon.
8. Involving consumer organizations (patients and family members) in the decision-making circle, as part of management teams in the areas, and as part of the therapeutic system (expansion of programs like “expert peers,” “consumer service providers,” “return home”).
9. Lastly, but by no means the least important: a necessary condition for the proposed structural change is the prior agreement of the main actors on stage: the Ministry of Health, the Ministry of Finance, the health funds, and the Ministry of Welfare. Based on past experience, each of these has the power to thwart a first-order change.

**Table 1:** Mapping mental health system problems to proposed solutions

Problem	Proposed Solution
Fragmented service delivery	Implement regional-integrational reform with comprehensive treatment zones (regional mental health centers)
Overreliance on hospital-based care	Transition to a community-based model with a continuum of care intensities
Lack of continuity in patient care	Introduce treatment coordinators to manage patient transitions across the care continuum
Insufficient early intervention	Develop specialized services for early diagnosis and intervention, especially for young people
Workforce shortages	Reverse the workforce pyramid, relying more on therapists with shorter training times for initial response and triage
Geographical disparities in access	Utilize technology to fill workforce gaps and address geographical distribution issues
Ineffective economic incentives	Implement value-based therapeutic compensation and incentives for community-based care
Limited patient and family involvement	Involve consumer organizations in decision-making and expand programs like “expert peers” and “consumer service providers”
Chronic underfunding	Increase public expenditure on mental health by 5 billion NIS over five years
Diagnostic limitations of current psychiatric categories	Shift focus from strict diagnostic categories to symptom clusters and functional assessments in line with the recovery model
Limited efficacy of current biological treatments	Emphasize psychosocial interventions and recovery-oriented care alongside pharmacological treatments
Lack of integration between mental health and primary care	Strengthen links between mental health services and primary care, potentially through shared care models
Insufficient focus on recovery and quality of life	Implement a recovery-based model focusing on personal goals, community integration, and overall well-being
Limited use of peer support	Expand peer support programs and integrate peer workers into mental health teams
Inadequate crisis intervention services	Develop community-based crisis intervention teams and improve access to crisis services
Lack of tailored approaches for different patient groups	Differentiate treatment approaches for SMI, non-SMI, and specific conditions like PTSD

### Public expenditure on mental health

Public expenditure on mental health, as we recently estimated [10], stands at about 4 billion NIS per year. The required programmatic change, as we propose here, has a clear economic price tag for the entire system. The addition will be front-loaded, with most expenditure in the early years to build services by the areas and recruit relevant manpower.

## Conclusion

These principles (brought here, of course, in summary) require further development and precise adaptation to the local Israeli system, but they address most of the fracture lines described above. Such a process is expected to create a less fragmented, more collaborative, more efficient (clinically and economically), integrative system that is ultimately more suited to future mental health demands in Israel. It should be noted that following the needs expected after the events of October, the Ministry of Health has already begun programs to change the existing system, some in the direction we outlined above. However, in our assessment, a broader vision for a more comprehensive plan is required, with significant depth changes and a future-oriented perspective for the coming years.

## Data Availability

Data sharing is not applicable to this article as no datasets were generated or analysed during the current study.

## Abbreviations

GDP: Gross domestic product
OECD: Organisation for economic co-operation and development
DSM: Diagnostic statistic manual
ICD: International classification of diseases
HMO: Health maintenance organisation
